# Exome Sequencing Reveals Primary Immunodeficiencies in Children with Community-Acquired *Pseudomonas aeruginosa* Sepsis

**DOI:** 10.3389/fimmu.2016.00357

**Published:** 2016-09-20

**Authors:** Samira Asgari, Paul J. McLaren, Jane Peake, Melanie Wong, Richard Wong, Istvan Bartha, Joshua R. Francis, Katia Abarca, Kyra A. Gelderman, Philipp Agyeman, Christoph Aebi, Christoph Berger, Jacques Fellay, Luregn J. Schlapbach, The Swiss Pediatric Sepsis Study, Klara Posfay-Barbe

**Affiliations:** ^1^Global Health Institute, School of Life Sciences, École Polytechnique Fédérale de Lausanne (EPFL), Lausanne, Switzerland; ^2^Swiss Institute of Bioinformatics, Lausanne, Switzerland; ^3^National HIV and Retrovirology Laboratory, Public Health Agency of Canada, Winnipeg, MB, Canada; ^4^Department of Medical Microbiology and Infectious Diseases, University of Manitoba, Winnipeg, MB, Canada; ^5^Lady Cilento Children’s Hospital, Brisbane, QLD, Australia; ^6^Children’s Hospital Westmead, Sydney, NSW, Australia; ^7^Pathology Queensland Central Laboratory, Royal Brisbane and Women’s Hospital, Brisbane, QLD, Australia; ^8^Menzies School of Health Research, Charles Darwin University, Darwin, NT, Australia; ^9^Royal Darwin Hospital, Darwin, NT, Australia; ^10^Departamento de Enfermedades Infecciosas e Inmunología Pediátrica, Escuela de Medicina, Pontificia Universidad Católica de Chile, Santiago, Chile; ^11^Sanquin Diagnostic Services, Amsterdam, Netherlands; ^12^Department of Pediatrics, Inselspital, Bern University Hospital, University of Bern, Bern, Switzerland; ^13^University Children’s Hospital Zurich, Zurich, Switzerland; ^14^Paediatric Critical Care Research Group (PCCRG), Mater Research, University of Queensland, Brisbane, QLD, Australia

**Keywords:** bacteremia, sepsis, child, *Pseudomonas*, primary immunodeficiency, exome sequencing

## Abstract

One out of three pediatric sepsis deaths in high income countries occur in previously healthy children. Primary immunodeficiencies (PIDs) have been postulated to underlie fulminant sepsis, but this concept remains to be confirmed in clinical practice. *Pseudomonas aeruginosa* (*P. aeruginosa*) is a common bacterium mostly associated with health care-related infections in immunocompromised individuals. However, in rare cases, it can cause sepsis in previously healthy children. We used exome sequencing and bioinformatic analysis to systematically search for genetic factors underpinning severe *P. aeruginosa* infection in the pediatric population. We collected blood samples from 11 previously healthy children, with no family history of immunodeficiency, who presented with severe sepsis due to community-acquired *P. aeruginosa* bacteremia. Genomic DNA was extracted from blood or tissue samples obtained intravitam or postmortem. We obtained high-coverage exome sequencing data and searched for rare loss-of-function variants. After rigorous filtrations, 12 potentially causal variants were identified. Two out of eight (25%) fatal cases were found to carry novel pathogenic variants in PID genes, including *BTK* and *DNMT3B*. This study demonstrates that exome sequencing allows to identify rare, deleterious human genetic variants responsible for fulminant sepsis in apparently healthy children. Diagnosing PIDs in such patients is of high relevance to survivors and affected families. We propose that unusually severe and fatal sepsis cases in previously healthy children should be considered for exome/genome sequencing to search for underlying PIDs.

## Introduction

Despite worldwide reduction in childhood mortality, sepsis remains one of the leading causes of childhood deaths. Importantly, 35–50% of pediatric sepsis deaths occur in previously healthy children (1–3). These children often develop non-specific symptoms suggestive of a viral respiratory infection, followed by sudden deterioration and rapid progression to shock and multisystem organ failure. In addition to pathogen virulence factors, such as streptococcal Toxic Shock Toxin (4), rare genetic variants causing a primary immunodeficiency (PID) may underlie fulminant sepsis. Such variants have high effect sizes and are usually kept at low frequencies in the population due to purifying selection (5, 6). Previous studies searching for PID in children with bacterial sepsis were limited to conventional immunological testing (5). Using high-throughput sequencing studies on individuals with extreme phenotypes, who are most likely to be informative from a genetic point of view, has been demonstrated to be very powerful in Mendelian diseases but has not been reported in patient cohorts with invasive bacterial infections (6).

*Pseudomonas aeruginosa* (*P. aeruginosa*) is an aerobic Gram-negative bacterium commonly found in environment. This opportunistic pathogen causes invasive infections in immunosuppressed and hospitalized patients and represents a major cause of health care-related infections. In contrast, sepsis due to community-acquired *P. aeruginosa* is extremely rare in apparently healthy children, and carries very high mortality (7, 8), and may be the first manifestation of an underlying PID. Indeed, a few case reports have already described the identification of PIDs in children with *P. aeruginosa* sepsis (9, 10). Here, we used exome sequencing and bioinformatic analysis to identify genetic variants conferring extreme susceptibility to *P. aeruginosa* in a cohort of 11 previously healthy children.

## Materials and Methods

### Patients

Children below 60 months with community-acquired blood-culture positive *P. aeruginosa* sepsis were eligible. Children with any comorbidities (prematurity, congenital malformations, previous surgery, immunosuppression, known immunodeficiency, and chronic diseases) and children who had been exposed to broad-spectrum intravenous antibiotics were excluded. This study was carried out in accordance with the recommendations of the ethics committees of participating centers (Kantonale Ethikkommission Bern, KEK Ref Nr 029/11, Bern, Switzerland; Mater Health Services HREC13/MHS/4, Brisbane, QLD, Australia). Parents/guardians of all included patients gave written informed consent in accordance with the Declaration of Helsinki. Patients were recruited prospectively and retrospectively in hospital databases and infectious diseases networks. Whenever possible, DNA was also collected from the parents.

### DNA Extraction and Exome Sequencing

Genomic DNA was extracted from whole blood (*N* = 8), frozen skin biopsy or lymphatic tissue (*N* = 2), or paraffin-fixed histology slides (*N* = 1). Exome sequencing libraries were prepared using Agilent SureSelect (V5, 50.4 Mb). Cluster generation was performed using Illumina TruSeq PE Cluster Kit v5 reagents. Libraries were sequenced as 100 bp long, paired-end reads on Illumina HiSeq 2500 using TruSeq SBS Kit v5 reagents.

### Short Read Alignment

Sequencing reads were processed using CASAVA v1.82. Reads were aligned to the human reference genome hg19 using BWA (11, 12) v0.6.2. PCR duplicates were removed using Picard 1.27-1 (http://picard.sourceforge.net/).

### Variant Calling

We used genome analysis toolkit (GATK) (13, 14) version 3.1-1 to call single nucleotide variants (SNVs) and small insertion and deletions (indels) from duplicate-marked bam files. We used HaplotypeCaller for multi-sample variant calling on all samples followed by GATK best practice to call the variants and included only variants that were flagged as PASS by GATK in subsequent analysis.

### Variant Effect Prediction

We used SnpEff (15) version 4.1B to predict the functional impact of variants. As variants can have several predicted effects, we only considered the most severe effect for each variant. The effects in decreasing order of severity are frameshift and in-frame for indels and stop-gain, splice site-disrupting, non-synonymous, synonymous, intronic, UTR, non-coding exon, and intergenic for SNVs. Variants were annotated as putative loss-of-function (LoF) if they were stop-gain or splice site-disrupting SNVs or frameshift indels mapping to the first 95% of coding region, or larger deletions removing either the first exon or more than 50% of the protein-coding sequence of the affected transcript.

### Variant Frequency Estimation

We checked the minor allele frequency (MAF) of all previously described variants in the following datasets: the NHLBI exome sequencing project (ESP, *N* = 6503) (16), the 1000 genomes project phase 2 (1KG, *N* = 876) (17) and the UK10K project (UK10K, *N* = 3621) (18), the Exome Aggregation Consortium database (ExAC, *N* = 60,706) (19), and a set of 533 in-house control exomes.

### Identification of Potentially Causal Variants in Parent–Child Trios

We restricted analyses to non-synonymous and LoF exonic variants with MAF <1% in ESP, 1KG, UK10K, ExAC, and in-house control exomes. We analyzed each family separately assuming autosomal recessive, autosomal dominant, and X-linked recessive inheritance models. Only variants with >10× coverage in both the parents and the offspring were included in the *de novo* analysis. We assumed full penetrance for the potentially causal variants and given the fatality of the phenotype did not consider a mutation as potentially causal if it was present in the parents, any of the abovementioned databases or in the in-house control exomes in the same zygosity form (i.e., heterozygous or homozygous) as in our patients.

### Identification of Potentially Causal Variants in Individual Patients

We restricted analyses to non-synonymous and LoF exonic variants with MAF <1% in ESP, 1KG, UK10K, ExAC, and in-house control exomes. We analyzed each individual separately assuming autosomal recessive and X-linked recessive inheritance models. We assumed full penetrance for the potentially causal variants and given the fatality of the phenotype did not consider a mutation as potentially causal if it was present in any of the abovementioned databases or in the in-house control exomes in the same zygosity form (i.e., heterozygous or homozygous) as in our patients.

### Targeted Search in Primary Immunodeficiency Genes

We ran a targeted search for rare (MAF <1%), non-synonymous, and LoF variants in a list of 252 known PID genes. This list includes known genes in 50 PID syndromes compiled by The International Union of Immunological Societies (IUIS) Expert Committee in 2015 (20) and 3 newly published PID genes since the latest release of IUIS till April 2016 (Table 1).

**Table 1:** **New PID genes discovered since the latest report of IUIS in 2015 till April 2016**.

Official gene name	Mutation	Inh.	Phenotype	Study population	Reference
STAT4	Missense	AD	Kaposi sarcoma	One consanguineous pedigree	(21)
MAP3K9	Non-sense	AR	Susceptibility to severe bacterial infection, *Pseudomonas* septic shock	One consanguineous pedigree	(22)
IRF3	Missense	AD	Herpes simplex encephalitis	16 sporadic cases	(23)

*AR, autosomal recessive; AD, autosomal dominant; Inh., inheritance; GOF, gain-of-function*.

### Gene-Annotation Enrichment Analysis

We used the database for annotation, visualization, and integrated discovery (DAVID) (24, 25) v6.7 with default options and highest classification stringency for functional annotation clustering of PID genes carrying rare, non-synonymous variants. We used hypergeometric test for assessing the significance of gene enrichment results.

### C9 Reconstitution Experiment

Serum of a patient heterozygous for a complement *C9* variant was diluted with GVB^++^ in a series starting at 1:10 to 1:640 in 1:2 steps. The same was done with a serum pool as a control. This pool consisted of serum from over 300 healthy donors. Forty microliters of these dilutions were pipetted in duplo in a round-bottom 96-wells plate. To these wells, 40 μl of GVB^++^ was added to the samples and the blanc, to the positive control 40 μl of 1.7% saponin was added (100% lysis). Ten microliters of purified C9 (Quidel) or GVB^++^ were added (end conc 50 μg/ml). Next, in all wells, 150 μl of EA’s were added. EA’s are sheep erythrocytes (Hatunalab, Sweden) coated with an optimal dose of Amboceptor (Rabbit-anti-Sheep erythrocyte; Dade Behring) brought to a concentration of 0.25 × 10^8^ cells/ml. This was incubated for 1 h at 37°C while agitating. After incubation, the plate was centrifuged for 5 min at 2000 rpm. Fifty microliters of each supernatant were pipetted to a flat-bottom 96-wells plate and diluted with 150 μl GVB^++^. Extinction was measured at 415 nm using a spectrophotometer. The percentage of lysis was calculated as follows:

((average of sample duplo−average blanc)/(average 100%−average blanc))×100.

GVB^++^ buffer consisted of 2 mM 5,5 di-ethylbarbituurzuur (Genfarma), 1.15 mM Na 5,5 di-ethylbarbituraat (Bufa), 96 mM NaCl (Merck), 0.5 mM CaCl (Merck), and 0.18 mM MgCl_2_ (Merck).

## Results

### Clinical Presentation

**Figure 1:**
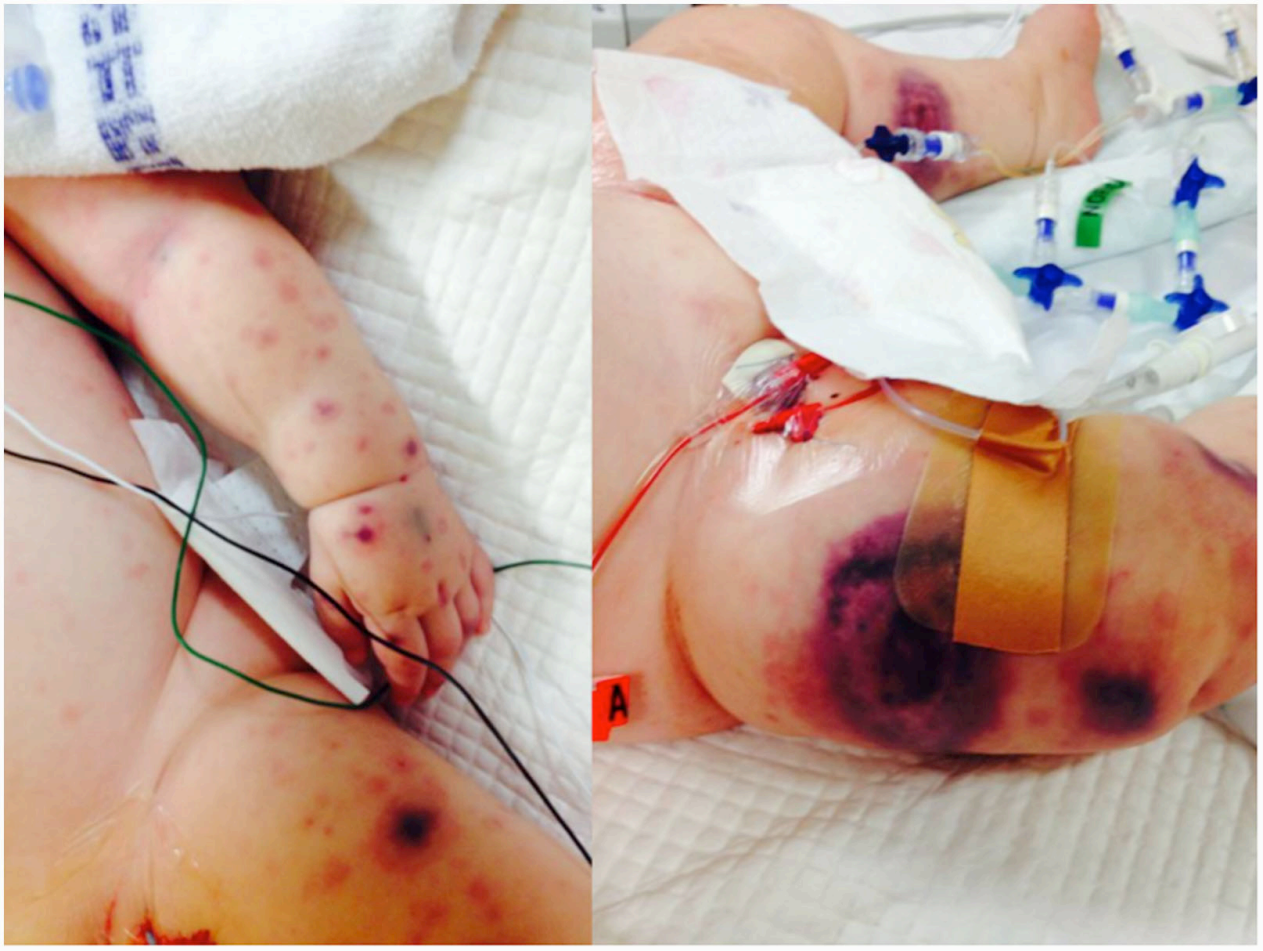
**Ecthyma gangrenosum, a highly suggestive rapidly progressive purpuric skin lesion seen in a minority of children with *P. aeruginosa* bacteremia, is shown in one of the study patients (with permission from parents)**.

We identified 11 previously healthy children with community-acquired *P. aeruginosa* bacteremia from whom DNA could be obtained. Child–parent DNA trios were available in seven affected families. The presenting age ranged from 6 months to 4 years, and 7/11 (64%) of patients were male (Table 2). All patients had never been admitted to hospital or exposed to intravenous antibiotics prior to presenting with *P. aeruginosa* sepsis. Nine patients had developed respiratory symptoms within 72 h of leading to hospital admission, one presented with Ecthyma gangrenosum (Figure 1), and eight (73%) died. Three children died in the emergency department shortly after presentation, and all deaths occurred within 48 h of hospital admission. Postmortem examination reports were available in seven cases. In four, *P. aeruginosa* grew in high quantities from nasal and oral swabs, tracheal secretions, and lungs. Viral co-infections were found in five deceased patients, including human herpes virus-6, parainfluenza virus-3, human metapneumovirus, respiratory syncytial virus, and varicella virus. One surviving patient presented with acute abdomen, and *P. aeruginosa* bacteremia was thought to result from intra-abdominal perforation. Another surviving patient was diagnosed with urosepsis and *P. aeruginosa* grew in urine and blood. The third surviving patient presented with recurrent parainfectious neutropenia. Six patients received aminoglycosides and/or anti-*Pseudomonas* beta-lactam antibiotics during sepsis, including all three survivors.

**Table 2:** **Demographic and clinical characteristics of included children with community-acquired *Pseudomonas aeruginosa* septicemia**.

Patient	DNA source	Phenotype	Parental DNA available	Ethnicity	Age (mo.)	Sex	Consanguinity	Viral co-infection	WCC	CRP	Clinical focus	Previous history
S1	Blood	Fatal	Yes	Caucasian	24	M	No	Parainflue nza 3	1.2	136	Pneumonia	Recurrent middle ear infections
S4	Blood	Fatal	Yes	Caucasian	13	M	No	HHV-6	0.6	NA	Septic shock	Recurrent middle ear infections
S7	Blood	Fatal	Yes	Caucasian	9	F	No	Parainflue nza 3	1.3	46	Ecthyma gangraenosum	Nil
S10	Blood	Survived	Yes	South American	8	M	No	None	2	132	Acute abdomen	Nil
S13	Blood	Fatal	No	Asian	26	M	Yes	Varicella	2.4	36	Septic shock	Recurrent respiratory infections
S14	Lymph node	Fatal	No	African	31	F	No	None	1	NA	Pneumonia	Nil
S15	Blood	Survived	No	Caucasian	56	M	No	None	26.9	235	Urosepsis	Nil
S16	Blood	Survived	No	Caucasian	7	M	No	None	1.2	301	Septic shock	Recurrent infection-associated neutropenia
S17	Fibroblasts	Fatal	Yes	Asian	9	M	No	hMPV, RSV	0.8	NA	Septic shock	Nil
S20	Paraffin slides	Fatal	Yes	Caucasian	30	M	No	None	1.2	213	Pneumonia	Recurrent respiratory infections
S23	Blood	Fatal	Yes	Asian	26	M	No	None	1.6	157	Septic shock	Recurrent respiratory infections

*F, female; M, male; mo, months old; CRP, C-reactive protein; WCC, white cell count (×10^9^/l)*.

### Exome Sequencing and Short Read Alignment

For each sample, 95% of reads passing filtering criteria were unique (not marked as duplicate); 97% of unique reads could be aligned to the human reference genome hg19. The mean on-bait coverage was 66×, with 95% of target bases achieving at least 10× coverage and 76% achieving at least 30× coverage (Table S1 in Supplementary Material). In 118 of the PID genes, at least one exon had an average coverage of <2×. Further investigation of these low-coverage intervals showed that the majority of them are overlapping with untranslated regions (UTRs).

### Variant Calling and Variant Annotation

A total of 115,604 SNVs and 10,814 indels passed GATK quality control, including 28,795 synonymous variants, 28,284 non-synonymous variants, 713 in-frame indels, 660 frameshift indels, 283 stop-gained, and 151 splice-site variants (Tables S2 and S3 in Supplementary Material).

### Variant Analysis

Thirty-eight rare, non-synonymous coding variants (MAF <1%) were observed in the 11 patients after testing for different inheritance models (Table 3), among which 12 were never seen in the same zygosity form (heterozygous or homozygous) in publicly available databases (Table 4). In addition to the above 38 variants, we performed a targeted search for rare, non-synonymous coding variants in 252 previously known PID genes and found 76 variants in 61 genes (Table 5). Functional classification of these 61 genes using DAVID highlighted the complement pathway as the most enriched cluster (DAVID enrichment score: 15.73). In total, 14 of the 28 complement pathway genes present in the list of 252 known PID genes carried at least one rare, non-synonymous coding variant in our cohort (hypergeometric probability: *p*(*x* > 14) = 0.003).

**Table 3:** **38 rare (MAF <1% in ESP, 1KP, UK10K, ExAC, and in-house controls) non-synonymous and putative LoF variants found in our 11 patients using different inheritance models (see Materials and Methods for details)**.

Patient	Inheritance	Chr	Position	Effect	Gene	ExAC MAF%
S4	AR	2	202957848	Missense	AC079354.1	0.33
S4	XL	X	99920649	Missense	SRPX2	0
S4	XL	X	100617192	Frameshift	BTK	0
S10	AD	13	112721975	Missense, *de novo*	SOX1	0
S10	AR	10	71906005	Missense	TYSND1	0.37
S10	XL	X	9864517	Missense	SHROOM2	0
S10	XL	X	53575033	Missense	HUWE1	0
S10	XL	X	70824419	Missense	ACRC	0
S13	AR	19	55879673	Missense	IL11	0.01
S13	AR	19	56423631	Missense	NLRP13	0.01
S13	AR	19	57132935	Missense	ZNF71	0
S13	AR	19	57840274	Missense	ZNF543	0.01
S13	AR	20	31765978	Missense	BPIFA2	0.02
S13	AR	20	31393172	In-frame indel	DNMT3B	0
S13	XL	X	53222222	Missense	KDM5C	1.41E−03
S13	XL	X	101619974	Missense	NXF2B	0
S14	AR	3	119526149	Missense	NR1I2	0.14
S14	AR	3	19977627	Splice site disrupting	EFHB	0.29
S14	AR	17	1265305	splice site disrupting	YWHAE	0.03
S14	AR	19	39362359	splice site disrupting	RINL	0.15
S15	XL	X	27998602	Missense	DCAF8L1	0.01
S15	XL	X	68382133	Missense	PJA1	0
S15	XL	X	101097713	Splice site disrupting	NXF5	0
S17	AR	1	6662288	Missense	KLHL21	0.16
S17	CH	1	155015918	Missense	DCST1	0.26
			155015948	Missense		0
S17	CH	10	112572308	Missense	RBM20	0.01
			112590912	Missense		0.02
S17	CH	17	76420087	Missense	DNAH17	0.01
			76522984	Missense		0.03
S17	XL	X	109561080	Missense	AMMECR1	0
S20	CH	19	41035017	Missense	SPTBN4	0.08
			41025445	Missense, *de novo*		0
S23	CH	16	4934707	Missense	PPL	8.23E−04
			4940241	Missense		0.05
S23	CH	17	28405505	Missense	EFCAB5	0.05
			28380755	Non-sense		0.01
S23	XL	X	19364694	Missense	PDHA1	0.29

*Chr, chromosome; AR, autosomal recessive; AD, autosomal dominant, XL, X-linked; CH, compound heterozygous*.

**Table 4:** **Potentially causal variants (see Materials and Methods for details) identified in 6 of the 11 previously healthy children with community-acquired *Pseudomonas aeruginosa* septicemia**.

Inheritance	Patient	Chr.	Position	Gene	Effect	ExAC MAF (%)
AR	S14	7	142562052	EPHB6	In-frame indel	0
	S13	20	31393172	DNMT3B	In-frame indel	0

AD	S20	19	41025445	SPTBN4	Missense, *de novo*	0
	S10	13	112721975	SOX1	Missense, *de novo*	0

XL	S4	X	100617192	BTK	Frameshift indel	0
	S10	X	27998602	DCAF8L1	Missense	1.14E−03
	S10	X	9864517	SHROOM2	Missense	0
	S10	X	53575033	HUWE1	Missense	0
	S10	X	70824419	ACRC	Missense	0
	S15	X	68382133	PJA1	Missense	0
	S15	X	101097713	NXF5	Splice site disrupting	0
	S15	X	109561080	AMMECR1	Missense	0

*Chr, chromosome; MAF, minor allele frequency; AR, autosomal recessive; AD, autosomal dominant, XL, X-linked*.

**Table 5:** **76 rare (ExAC MAF <1%) non-synonymous and putative LoF variants found in known 252 PID genes in our 11 patients**.

Patient	Chr.	Position	Effect	Type	Gene	ExAC MAF%
S1	11	108098576	Missense	Het	ATM	0.74
S10	9	340168	Missense	Het	DOCK8	0
S10	11	4104647	Missense	Het	STIM1	8.25E−04
S10	11	108117787	Missense	Het	ATM	0.13
S10	5	41155088	Missense	Het	*C6	0.69
S13	20	31393172	In-frame indel	Hom	DNMT3B	0
S13	8	100844596	Splice site disrupt	Het	VPS13B	0.43
S13	2	231036831	Missense	Het	SP110	4.12E−03
S13	5	77334907	Missense	Het	AP3B1	0.08
S13	6	31915584	Missense	Het	*CFB	0.1
S13	11	6637588	Missense	Het	TPP1	0.18
S13	11	2407334	Missense	Het	CD81	0.56
S13	5	147475388	Missense	Het	SPINK5	0.65
S13	5	35876300	Missense	Het	IL7R	0.24
S13	12	110034347	Missense	Hom	MVK	0.13
S13	1	196799796	Missense	Hom	*CFHR1	0.14
S13	10	6063567	Missense	Het	IL2RA	0.88
S14	8	100654621	Missense	Het	VPS13B	0.02
S14	12	110017618	Missense	Het	MVK	0.07
S14	8	100861113	Missense	Het	VPS13B	0.06
S14	1	11094908	Missense	Het	*MASP2	0.08
S14	15	91337505	Missense	Het	BLM	0.1
S14	8	48733399	Missense	Het	PRKDC	0.09
S14	6	32798457	Missense	Het	TAP2	0.49
S14	1	949422	Missense	Het	ISG15	0.16
S14	11	108129778	Missense	Het	ATM	0.21
S14	11	108123551	Missense	Het	ATM	0.29
S14	3	196198925	Missense	Het	RNF168	0.2
S14	8	90982691	Missense	Het	NBN	0.26
S14	9	139840153	Missense	Het	*C8G	0.46
S14	4	187004767	Missense	Het	TLR3	0.3
S14	6	32800427	Missense	Het	TAP2	0.61
S14	1	196684855	Missense	Het	*CFH	0.5
S14	10	73103969	Missense	Het	SLC29A3	0.54
S14	9	123751873	Missense	Het	*C5	0.61
S14	9	123737145	Missense	Het	*C5	0.91
S14	1	196715063	Missense	Het	*CFH	0.97
S14	20	62324328	Missense	Het	RTEL1	0.98
S15	5	39342214	Non-sense	Het	*C9	0.1
S15	2	47277182	Missense	Het	TTC7A	0.2
S15	2	47273468	Missense	Het	TTC7A	0.21
S15	10	97983635	Missense	Het	BLNK	0.55
S15	11	108138003	Missense	Het	ATM	0.91
S16	16	27460420	Missense	Het	IL21R	0.03
S16	9	139264888	Missense	Het	CARD9	0.35
S16	5	158750329	Missense	Het	IL12B	0.65
S17	17	73826517	Missense	Het	UNC13D	2.25E−03
S17	1	949431	Missense	Het	ISG15	4.17E−03
S17	16	50745960	Missense	Het	NOD2	0.03
S17	5	1268697	Missense	Het	TERT	0.09
S17	17	26875685	Missense	Het	UNC119	0.01
S17	17	76120792	Missense	Het	TMC6	0.65
S17	15	91295110	Missense	Hom	BLM	0.86
S17	8	42177163	Missense	Het	IKBKB	0.89
S20	11	118898444	Missense	Het	SLC37A4	0
S20	12	122064747	Missense	Het	ORAI1	0
S20	22	36662063	Missense	Het	APOL1	0.01
S20	1	22965341	Missense	Het	*C1QA	0.04
S20	11	108119823	Missense	Het	ATM	0.22
S20	4	151242409	Missense	Het	LRBA	0.46
S23	1	207646266	Missense	Het	*CR2	3.30E−03
S23	1	235896980	Missense	Het	LYST	0.02
S23	1	154247666	Missense	Het	HAhet	0.03
S23	1	183536358	Missense	Het	NCF2	0.23
S23	6	137540425	Missense	Het	IFNGR1	0.14
S23	22	31007023	Missense	Het	TCN2	0.27
S23	1	207925595	Missense	Het	*CD46	0.5
S23	5	40945397	Missense	Het	*C7	0.95
S4	X	100617192	Frameshift indel	Het	BTK	0
S4	1	151316324	Missense	Het	RFX5	0.88
S7	12	133263886	Missense	Het	POLE	0.14
S7	19	18170874	Missense	Het	IL12RB1	0.17
S7	X	77150892	Missense	Het	MAGT1	0.29
S7	16	81957106	Missense	Het	PLCG2	0.13
S7	4	110667485	Missense	Het	*CFI	0.34
S7	5	41155088	Missense	Het	*C6	0.69

*The genes marked by the asterisk are involved in complement activation*.

*Chr, chromosome; hom, homozygous; het, heterozygous*.

In one patient (patient S4) who died of fulminant septic shock, we identified a novel single-base insertion on the X chromosome (C > CT, chromosome 20, position 100617191) leading to a frameshift in the Bruton agammaglobulinemia tyrosine kinase (*BTK*) gene. Other than a middle ear infection during infancy, the previous history, growth, development, and vaccination history of the patient had been unremarkable. Taqman genotyping and clinical genetic testing confirmed presence of mutation in the deceased patient and revealed a *de novo* occurrence in the mother, while other family members were healthy. BTK plays a crucial role in B-cell development. Postmortem serological testing confirmed the absence of immunoglobulins, consistent with BTK LoF: IgG < 1.3 g/l (4.22–11.9); IgA < 0.2 g/l (0.2–1.58); IgM < 0.2g/l (0.48–1.9).

A novel homozygous deletion spanning 6 bp on chromosome 20 (ACTCGAG > A, X chromosome, position 31393171) was observed in a 2-year-old boy with consanguineous parents (patient S13), leading to an in-frame deletion in a conserved region of the catalytic domain of the *DNMT3B* gene. Previously described missense mutations in the same protein domain are known to cause immunodeficiency-centromeric instability-facial anomalies (ICF) syndrome (26), a rare disease characterized by variable immunodeficiency and recurrent infections with mild facial abnormalities. The fatal septicemia was preceded by uncomplicated varicella, and the patient had a history of recurrent mild respiratory and upper airway infections that had been attributed to poor health conditions. The patient had normal T and B cell counts but reduced immunoglobulin levels, consistent with ICF syndrome: IgG 0.16 g/l (4.22–11.9); IgA <0.06 g/l (0.2–1.58); IgM 0.05 g/l (0.48–1.9).

**Figure 2:**
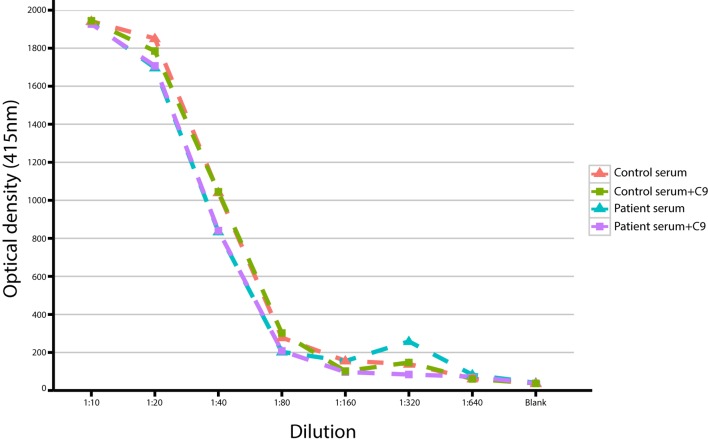
**Titration of serum with/without C9 in a study patient with a heterozygous stop-gain variant in the *C9* gene**.

We also observed a rare, known (MAF = 0.001 in ExAC) heterozygous stop-gain variant in the complement *C9*, on chromosome 5 (G > T, chromosome 5, position 39342214) in a male patient (patient S15) with severe *Pseudomonas* septicemia and recurrent parainfectious neutropenia. The patient survived without sequelae. Complement reconstitution assay with classical or terminal pathway proteins (C1–C9) showed normal lytic activity (Figure 2), suggesting that this variant is unlikely to be causal to increased susceptibility to *P. aeruginosa*.

## Discussion

We studied *P. aeruginosa* sepsis in previously healthy children as a model disease defining an extreme phenotype of fulminant sepsis. Fulminant sepsis in children is associated with high mortality, and the majority of deaths occur within hours of presentation (27). The disease burden is highest in children under 5 years of age (3, 7). We hypothesized that rare genetic variants with high effect sizes could explain life-threatening susceptibility to *P. aeruginosa* in children without known risk factors or comorbidities. Mendelian disorders of immunity have previously been shown to result in phenotypes with unusual susceptibility to bacterial infection, such as pseudomonal and pneumococcal infections in patients with IRAK-4 deficiency (10, 28). We used exome sequencing to explore the genetic cause of susceptibility to *P. aeruginosa* in a carefully selected group of previously healthy children with no familial history of immunodeficiency, who developed community-acquired *P. aeruginosa* septicemia. By systematic search for fully penetrant, rare, non-synonymous, and LoF variants in exonic regions, we identified 12 potentially causal variants including two novel variants in known PID genes: X-linked agammaglobulinemia due to a novel *BTK* mutation and ICF immunodeficiency syndrome due to a novel *DNMT3B* mutation. In addition, we performed a targeted search for rare, non-synonymous, and LoF variants in known PID genes and observed an enrichment of such variants among genes that are part of the complement pathway. One patient carried a heterozygous, stop-gain variant in the *C9* gene, which has been associated with recurrent meningitis (29, 30). However, functional testing of patient samples showed normal lytic activity, demonstrating the need for strict validation of potentially causal mutations, even in genes with plausible biological links to the study phenotype. Variants conferring life-threatening susceptibility to common infections in children below reproductive age are subjected to strong purifying selection and will therefore only be found at very low frequencies (31–33). Out of the 12 potentially causal variants described in this study, 11 were novel and 1 was a hemizygous variant present in 1 individual (in heterozygous form) in ExAC.

Previous case reports describing *P. aeruginosa* sepsis in children with known PIDs, such as Wiskott–Aldrich syndrome, X-linked agammaglobulinemia, cyclic neutropenia, and IRAK-4/MyD-88 deficiency (8, 10, 22, 34), were based on conventional, candidate–gene-driven immunological testing. Ecthyma gangrenosum is present in a minority of patients and has been described as the presenting sign of an underlying PID, such as cyclic neutropenia, chronic granulomatous disease, or hypogammaglobulinemia (35–37). The range of PID types identified in these previous reports indicates that multiple distinct genetic defects have to be considered when investigating such clinical presentations. Importantly, classic PID screening may fail to identify new mutations in known PID genes (38) and will miss causal mutations in genes without an established link with PIDs. Failure to diagnose rare underlying PID in a child presenting with sepsis may result in potentially devastating consequences for survivors, undiagnosed siblings, and their families. High-throughput sequencing, in particular exome sequencing, has proved highly successful in the clinical diagnosis of suspected monogenic conditions in the pediatric population (39–41). This was recently illustrated by Record et al., who used exome sequencing in a girl with *P. aeruginosa* sepsis and found a homozygous LoF mutation in *MAP3K9* (*MKL1*), a gene that was not known to cause PID (22).

A strength of the present cohort is that the case definition required significant growth of community-acquired *P. aeruginosa* in blood culture in previously healthy children presenting with signs and symptoms of sepsis, thereby constituting an extreme phenotype. The majority of children included in this study presented with fulminant sepsis and septic shock due to *Pseudomonas* sepsis. In the cases where postmortem examinations were performed, extensive growth of *P. aeruginosa* was found in several tissues, suggesting overwhelming bacterial infection. In agreement with other studies, we observed that several sepsis patients had viral co-infections (3). The role of viral co-infections in the pathogenesis of childhood bacterial sepsis is poorly understood and may include facilitated bacterial invasion due to respiratory epithelial disruption and increased host susceptibility during viremia (42). Immunological investigations in children presenting with fulminant sepsis can be extremely challenging, as severe leukopenia, coagulopathy, multi-organ failure, and fluid resuscitation often lead to severe alterations of cell counts, immunoglobulin levels, and complement levels. In addition, a proportion of patients die prior admission to an intensive care unit, or present with out-of-hospital cardiac arrest or sudden infant death syndrome (43). To date, there are no widely accepted guidelines to inform pediatricians, emergency and intensive care physicians, and pathologists about indications for specific immunological investigations in previously healthy children presenting with fulminant or fatal sepsis (44). Given the high fatality in our cohort, with several cases recruited considerable time after death of the patient, our study highlights the value of performing exome sequencing in this population using blood or tissue containing DNA. However, the lack of viable host cells in deceased patients may represent a major limitation toward functional validation of potentially causal variants. Elucidating the role of these variants in novel genes that may confer a PID phenotype will therefore require independent validation in other patients or cohorts.

While it is well known that PIDs can be responsible for severe bacterial infections, little is known about the proportion of children with invasive infections suffering from PID (5). In our cohort, defects in known PID genes were found in 25% of fatal cases. This is comparable to a recent study using exome sequencing in 50 patients with common variable immunodeficiency where exome sequencing identified disease-causing mutations in 30% of cases (45). While non-genetic factors, including variation in pathogen virulence and secondary neutropenia, may be responsible for the remaining cases, we cannot rule out that mutations in genes not previously associated with PID are at least partially involved. Sequencing of additional family members, sequencing of more cases with the same phenotype, and functional characterization of new candidate genes and variants will be required to identify such genetic factors. Furthermore, whole-genome sequencing is needed to explore the non-coding variants, large structural variants, and also exonic variants that are not be well-covered using exome sequencing.

In conclusion, this study provides proof of concept that exome sequencing allows the identification of rare genetic variants responsible for fulminant sepsis in children in whom immunodeficiency had not been previously suspected. Given the decreasing cost of exome and genome sequencing (39, 40, 46), we propose considering host DNA sequencing as a possible diagnostic procedure for apparently healthy children presenting with unusually severe or fatal sepsis.

## Author Notes

Swiss Pediatric Sepsis Study Group: Klara Posfay-Barbe, Department of Pediatrics, University Hospitals of Geneva; Eric Giannoni, Service of Neonatology, Lausanne University Hospital, University of Lausanne, Lausanne; Christoph Aebi, Philipp Agyeman, Bendicht P. Wagner, and Luregn J. Schlapbach, Department of Pediatrics, Inselspital, Bern University Hospital, University of Bern, Switzerland; Ulrich Heininger, Infectious Diseases and Vaccinology, University of Basel Children’s Hospital, Basel; Gabriel Konetzny, Children’s Hospital Aarau; Alex Donas, Martin Stocker, Children’s Hospital Lucerne; Antonio Leone, Paul Hasters, Department of Neonatology, University Hospital Zurich; Anita Niederer-Loher, Christian Kahlert, Children’s Hospital of Eastern Switzerland St. Gallen; Walter Baer, Children’s Hospital Chur; Christa Relly, Christoph Berger, University Children’s Hospital Zurich, Switzerland.

## Author Contributions

LS was responsible for the design of the study, patient recruitment and data acquisition, analysis, interpretation, and drafting of the manuscript. SA and JF were involved in the study design, genomic analyses, interpretation, and drafting of the manuscript. PM and IB were involved in genomic analyses, helped revise the manuscript, and approved the final version of the manuscript. JP, MW, RW, JRF, KA, KG, PA, CA, and CB were involved in study design, performed patient recruitment, were involved in revising the manuscript, and approved the final version.

## Conflict of Interest Statement

The authors declare that the research was conducted in the absence of any commercial or financial relationships that could be construed as a potential conflict of interest.
