# Corrigendum: Exome Sequencing Reveals Primary Immunodeficiencies in Children with Community-Acquired *Pseudomonas aeruginosa* Sepsis

**DOI:** 10.3389/fimmu.2016.00447

**Published:** 2016-10-27

**Authors:** Samira Asgari, Paul J. McLaren, Jane Peake, Melanie Wong, Richard Wong, Istvan Bartha, Joshua R. Francis, Katia Abarca, Kyra A. Gelderman, Philipp Agyeman, Christoph Aebi, Christoph Berger, Jacques Fellay, Luregn J. Schlapbach, The Swiss Pediatric Sepsis Study

**Affiliations:** ^1^Global Health Institute, School of Life Sciences, École Polytechnique Fédérale de Lausanne (EPFL), Lausanne, Switzerland; ^2^Swiss Institute of Bioinformatics, Lausanne, Switzerland; ^3^National HIV and Retrovirology Laboratory, Public Health Agency of Canada, Winnipeg, MB, Canada; ^4^Department of Medical Microbiology and Infectious Diseases, University of Manitoba, Winnipeg, MB, Canada; ^5^Lady Cilento Children’s Hospital, Brisbane, QLD, Australia; ^6^Children’s Hospital Westmead, Sydney, NSW, Australia; ^7^Pathology Queensland Central Laboratory, Royal Brisbane and Women’s Hospital, Brisbane, QLD, Australia; ^8^Menzies School of Health Research, Charles Darwin University, Darwin, NT, Australia; ^9^Royal Darwin Hospital, Darwin, NT, Australia; ^10^Departamento de Enfermedades Infecciosas e Inmunología Pediátrica, Escuela de Medicina, Pontificia Universidad Católica de Chile, Santiago, Chile; ^11^Sanquin Diagnostic Services, Amsterdam, Netherlands; ^12^Department of Pediatrics, Inselspital, Bern University Hospital, University of Bern, Bern, Switzerland; ^13^University Children’s Hospital Zurich, Zurich, Switzerland; ^14^Paediatric Critical Care Research Group (PCCRG), Mater Research, University of Queensland, Brisbane, QLD, Australia

**Keywords:** bacteremia, sepsis, child, *Pseudomonas*, primary immunodeficiency, exome sequencing

There was a mistake in the gene name reference to MKL1 on page 6, right column, last sentence of 2nd paragraph. The sentences says: “This was recently illustrated by Record et al., who used exome sequencing in a girl with *P. aeruginosa* sepsis and found a homozygous LoF mutation in MAP3K9 (MKL1), a gene that was not known to cause PID (22).” The correct form of the sentence should be “This was recently illustrated by Record et al., who used exome sequencing in a girl with *P. aeruginosa* sepsis and found a homozygous LoF mutation in MKL1, a gene that was not known to cause PID (22).”

The authors apologize for the mistake.

This error does not change the scientific conclusions of the article in any way.

## Conflict of Interest Statement

The authors declare that the research was conducted in the absence of any commercial or financial relationships that could be construed as a potential conflict of interest.

